# Editorial: Advances in Multi-Scale Analysis of Brain Complexity

**DOI:** 10.3389/fnins.2020.00337

**Published:** 2020-04-15

**Authors:** Albert C. Yang, Kay Jann, Christoph M. Michel, Danny J. J. Wang

**Affiliations:** ^1^Division of Interdisciplinary Medicine and Biotechnology, Beth Israel Deaconess Medical Center, Harvard Medical School, Boston, MA, United States; ^2^Digital Medicine Center, Institute of Brain Science, National Yang-Ming University, Taipei, Taiwan; ^3^Laboratory of Functional MRI Technology, Keck School of Medicine, Stevens Neuroimaging and Informatics Institute, University of Southern California, Los Angeles, CA, United States; ^4^Functional Brain Mapping Lab, Department of Fundamental Neurosciences, University of Geneva, Geneva, Switzerland

**Keywords:** brain, complexity, EEG, fMRI — functional magnetic resonance imaging, multiscale (MS) modeling

In neuroscience, a defining but elusive question regarding the human brain is its astonishingly structural and functional complexity. This complexity arises from the interaction of numerous neuronal circuits that operate over a wide range of temporal and spatial scales, enabling the brain to adapt to the constantly changing environment and to perform various high-level mental functions. Such dynamical and functional adaptability is often reduced during the aging process and considerably impaired in patients with neuropsychiatric diseases, leading to rigid, fixed, or on the opposite, unpredictable behaviors. Recently, attempts have been made to apply concepts adopted from complexity science to more fully understand complex brain functions as indicated by the signals from the brain and their implications on human behavior. Therefore, this Research Topic, “Advances in Multi-Scale Analysis of Brain Complexity,” is devoted to the research of brain complexity at multiple spatial and temporal scales and its role in neuropsychiatric diseases.

In this Research Topic, a significant portion of focus was on the multi-scale analyses of brain complexity in Alzheimer's disease (AD), which is a progressive brain disorder with gradual memory loss that correlates to cognitive deficits in the elderly population. Importantly, the complexity analysis of brain signal, such as an electroencephalography (EEG), could be a marker for disease severity. Fan et al. applied machine learning algorithms to classify the different stages of AD using the complexity analysis of scalp EEG signals with an accuracy of 80% in differentiating normal and mild cognitive impairment (MCI). The study further found that temporal and occipitoparietal brain regions were more discriminative with regard to classifying severe AD cohort vs. normal controls, which could be a marker for evaluating the severity of AD (Fan et al.). Niu et al. applied multiscale entropy analysis in resting-state fMRI data from the Alzheimer's disease neuroimaging initiative database. They found that both MCI and AD patients had significant reductions in the complexity of resting-state fMRI signals compared to healthy controls, and AD patients also demonstrated lower complexity than that of the MCI subjects (Niu et al.). Grieder et al. investigated alterations of functional connectivity and brain signal complexity within the default mode network (DMN) in 15 mild Alzheimer's disease patients as compared to 14 controls. Their findings suggested that cognitive decline in Alzheimer's disease is reflected by decreased brain signal complexity in DMN nodes, which might further lead to disrupted DMN functional connectivity (Grieder et al.). These findings support the notion that the multi-scale analysis of brain signal complexity may provide a functional or imaging marker of cognitive impairments in the neurodegenerative disease. Interestingly, Eagleman et al. applied brain signal complexity using 1/f power law scaling as well as measures of complexity from non-linear dynamics in geriatric patients that carry increased risk for adverse cognitive outcomes after anesthesia. This study found that both spectral and complexity measures are capable of capturing subtle differences in EEG activity with anesthesia administration—differences which future work may reveal to improve geriatric patient monitoring (Eagleman et al.).

Another focus of this Research Topic was in the developing brain. Hasegawa et al. used magnetoencephalography (MEG) to study special populations, the infant. Their analyses revealed time scale-dependent developmental trajectories based on MEG signal complexity. Specifically, MEG signal complexity predominantly increased from 5 to 15 months of age at higher temporal scales, whereas the complexity at lower temporal scales was constant across age, except in one infant who showed decreased complexity. The results of this pilot study may serve to further our understanding of the longitudinal changes in the neural dynamics of the developing infant brain (Hasegawa et al.). On the other hand, Smith et al. examined the relationship between the resting-state networks entropy and integrity in patients with autism spectrum disorder (ASD) and typically developing (TD) individuals from the Autism Brain Imaging Data Exchange (ABIDE) cohort. They found that complexity of resting-state fMRI signal within resting-state networks significantly distinguished ASD from TD, and the level of brain signal complexity was associated with ASD symptom severity. Importantly, they found that imbalanced brain connectivity and dynamics at the network level coincides with their decoupling in ASD, suggesting a link between changes in brain signal dynamics and network decoupling in the pathologic conditions (Smith et al.). Furthermore, Hager et al. explored how non-linear brain dynamics change during motor resonance, which is often used to study social interaction deficiencies in ASD. This paper performed an elegant experiment in an adult population and found that the desynchronization of the mu wave during motor resonance results in a local increase of mu entropy in sensorimotor areas, potentially reflecting a release from alpha inhibition. This release from inhibition may be mediated by the baseline complexity in the mu band. These findings suggest that dynamical complexity and network analysis of EEG may provide a useful addition for future studies of motor resonance by incorporating measures of non-linearity (Hager et al.).

Brain signal complexity is also implicated in sleep physiology. Hou et al. investigated whether lower complexity of brain waves at the pre-sleep state can facilitate sleep initiation and further improve sleep quality. The study based on polysomnographic recordings from Sleep Heart Health Study identified that lower complexity before sleep onset is associated with decreased sleep latency, indicating a potential facilitating role of reduced pre-sleep complexity in the wake-sleep transition (Hou et al.).

Additionally, there are increasing interests in studying brain signal complexity and its relationship with genetic polymorphisms. For example, brain-derived neurotrophic factor (BDNF) is a widely expressed neurotrophin in the brain and is crucial to neural plasticity. The BDNF Val66Met single-nucleotide polymorphism is associated with mood, stress, and pain conditions. In this Research Topic, Low et al. found that brain complexity alterations were associated with the interactions of BDNF Val66Met polymorphism and menstrual pain experience, suggesting that pain experience preponderantly affects the effect of BDNF Val66Met polymorphism on brain complexity.

An important, yet overlooked issue in non-linear analysis of brain signal dynamics is the choice of parameters in these non-linear methods. For example, there are several parameters that need to be determined when estimating the entropy of brain signal, and the choice of parameter may affect the reliability of entropy estimates. Yang et al. illustrate a general strategy for selecting entropy parameters to reduce the bias of entropy estimates in resting-state fMRI signals. In this paper, they present a minimizing error approach to reduce the bias of entropy estimates in resting-state fMRI data. The strategy explored a range of parameters that minimized the relative error of entropy of resting-state fMRI signals in cerebrospinal fluids where minimal physiologic information was present, and applied these parameters to calculate entropy of resting signals in gray matter regions. This strategy may help minimize the error of entropy estimates for future studies on the non-linear analysis of relatively short resting-state fMRI signals (Yang et al.).

Finally, an important question is: what are the physiologic mechanisms of brain signal complexity? Billings et al. proposes to represent resting-state fMRI signal as multiple processes occurring over multiple time scales, which could potentially implicate in the physiological mechanisms of brain signal complexity. Importantly, Wang et al. investigated the neurophysiological underpinnings of the complexity of electrophysiology and resting-state fMRI signals and their relations to functional connectivity. They found that regional neural complexity and network functional connectivity may be two related aspects of the brain's information processing—the more complex the regional neural activity, the higher functional connectivity this region has with other brain regions. Wang et al. propose that the complexity of regional neural signals may serve as an index of the brain's capacity of information processing—increased complexity may indicate greater transition or exploration between different states of brain networks, thereby indicating a greater propensity for information processing.

Overall, this Research Topic focuses on the theoretic and quantitative analysis of brain complexity in normal mental functions, as well as how it changes with neuropsychiatric diseases by using electrophysiological recordings or functional brain imaging data. These studies can provide a novel computational approach for extracting the fundamental features of the human brain. We anticipate that these approaches will provide better characterization of the heterogeneity of neuropsychiatric diseases and will highlight a subset of dynamical brain markers that will lead to translational research utilizing these complexity methods for understanding complex brain functions.

## Author Contributions

AY, KJ, CM, and DW read and approved the editorial.

### Conflict of Interest

The authors declare that the research was conducted in the absence of any commercial or financial relationships that could be construed as a potential conflict of interest.

